# Efficacy of cognitive behavioral therapy for smoking cessation: A systematic review and meta-analysis

**DOI:** 10.18332/tid/225228

**Published:** 2026-07-22

**Authors:** Yohei Okawa

**Affiliations:** 1 Graduate School of Nursing Science, St. Luke's International University, Tokyo, Japan

**Keywords:** cognitive behavioral therapy, smoking cessation, tobacco use cessation, systematic review, meta-analysis

## Abstract

**INTRODUCTION:**

Tobacco use is a major preventable cause of morbidity and mortality, and Cognitive Behavioral Therapy (CBT) may address the cognitive, behavioral, and relapse-related mechanisms that sustain smoking. This review evaluated the efficacy of CBT for smoking cessation by synthesizing controlled studies reporting smoking abstinence and smoking reduction outcomes.

**METHODS:**

Within this systematic review and meta-analysis, a search was performed in PubMed, Web of Science, Scopus, ScienceDirect, SpringerLink, ACM Digital Library, IEEE Xplore, and Google Scholar for studies published from 1 January 2000 to 15 January 2026. Eligible studies were randomized controlled trials or controlled quasi-experimental studies of people who smoked and compared CBT, delivered face-to-face or remotely, with a non-CBT control condition. Outcome data were extracted for smoking abstinence and smoking reduction. Risk-of-bias was assessed using RoB 2 for randomized trials and ROBINS-I for non-randomized controlled studies. Odds Ratios (ORs) were pooled using DerSimonian and Laird random-effects models, with fixed-effect estimates used only as sensitivity analyses.

**RESULTS:**

Fourteen studies were included. Eleven studies contributed to the smoking abstinence meta-analysis, and five contributed to the smoking reduction meta-analysis. For smoking abstinence, the random-effects pooled OR was 1.60 (95% CI: 0.95–2.70; z=1.76; p=0.078; I^2^=83.70%). For smoking reduction, the random-effects pooled OR was 1.96 (95% CI: 0.66–5.81; z=1.21; p=0.226; I^2^=83.64%). Risk-of-bias assessments indicated low-risk, some-concerns, or moderate-risk ratings across included studies; no included study was judged to have a critical risk-of-bias.

**CONCLUSIONS:**

CBT showed a favorable direction of effect for smoking abstinence, but the random-effects estimate was imprecise, and heterogeneity was substantial. Evidence for smoking reduction was also not statistically conclusive. The findings should therefore be interpreted cautiously, and future trials should clarify treatment moderators, standardize outcome definitions, and refine CBT protocols for different cessation goals.

## INTRODUCTION

Tobacco use remains a major preventable cause of morbidity and mortality worldwide, with recent global health estimates attributing more than seven million deaths each year to tobacco^1^. Nicotine dependence is sustained by interacting biological, psychological, behavioral, and social processes, and many people who smoke relapse despite pharmacological support such as nicotine replacement therapy, varenicline, or bupropion^2,3^. Psychological interventions are therefore important because they can address beliefs, routines, cues, and coping behaviors that maintain tobacco use.

Cognitive Behavioral Therapy (CBT) is one of the most established psychological approaches to smoking cessation. It typically aims to help individuals identify smoking-related thoughts and triggers, develop alternative coping strategies, manage cravings and withdrawal symptoms, and prevent relapse^4,5^. Controlled trials have compared CBT with usual care, brief advice, supportive counseling, pharmacotherapy alone, and other control conditions, but their findings have been inconsistent^5,6^. This inconsistency may reflect variation in CBT intensity, delivery format, population characteristics, and outcome definitions^7-9^.

Previous reviews have provided important summaries of behavioral interventions for tobacco cessation, but many have focused on specific populations or have combined CBT with other behavioral approaches, making it difficult to isolate the contribution of CBT^6-8^. In addition, complete abstinence and smoking reduction are often treated as related outcomes, although they may require different therapeutic mechanisms. The present systematic review and meta-analysis, therefore, aimed to estimate the pooled effect of CBT on two outcomes: smoking abstinence and smoking reduction.

## METHODS

### Review protocol and search strategy

This systematic review and meta-analysis were conducted in accordance with the Preferred Reporting Items for Systematic Reviews and Meta-Analyses (PRISMA) 2020 statement^10^. PubMed, Web of Science, Scopus, ScienceDirect, SpringerLink, ACM Digital Library, IEEE Xplore, and Google Scholar were searched to identify relevant studies. The search was conducted on 15 January 2026 and covered publications from 1 January 2000 to the search date. The date restriction was selected to focus on contemporary CBT protocols, and outcome definitions used after the widespread adoption of modern evidence-based cessation services; older trials were considered less comparable with contemporary pharmacotherapy and behavioral-support contexts. The complete database-specific search strings are presented in Supplementary file Table 1.

Records were imported into a reference-management spreadsheet, and automated duplicate detection based on title, author, year, and digital object identifier was followed by manual verification. The screening and extraction procedures were conducted by the author; because a second independent reviewer was not available, borderline decisions were checked twice against the eligibility criteria before final inclusion.

### Eligibility criteria

Studies were eligible if they were randomized controlled trials or controlled quasi-experimental studies, included people who smoked, and evaluated CBT as a primary or major intervention component. CBT could be delivered individually, in groups, digitally, or through telehealth. Eligible comparators included no treatment, waitlist, usual care, brief advice, pharmacotherapy alone, or another psychological intervention that did not include CBT as its main component.

Studies had to report at least one smoking cessation outcome, including point-prevalence, continuous or prolonged abstinence, biochemically verified abstinence, or clinically meaningful smoking reduction. Non-original reports, single-arm studies, case reports, qualitative studies, protocols without results, dissertations, conference abstracts, preprints, and grey literature were excluded. Studies were also excluded if CBT was combined with another intensive psychotherapy in a way that prevented isolation of the CBT effect, if no non-CBT control group was available, if follow-up was shorter than four weeks after the quit date, or if a study had a high or critical risk of bias in key domains such as allocation, confounding, attrition, or outcome reporting.

### Risk-of-bias assessment

Risk-of-bias was assessed using the revised Cochrane Risk-of-Bias 2 tool (RoB 2) for randomized trials and ROBINS-I for non-randomized controlled studies^11,12^. RoB 2 domains included bias arising from the randomization process, deviations from intended interventions, missing outcome data, outcome measurement, and selection of the reported result. ROBINS-I domains were summarized by focusing on confounding, selection, classification of interventions, deviations from intended interventions, missing data, outcome measurement, and selection of reported results. Overall judgments were classified as low risk, some concerns, or high risk for randomized trials, and as low, moderate, serious, or critical risk for non-randomized studies. Studies judged to have high or critical risk in key domains were excluded.

### Study selection and data extraction

For each eligible study, data were extracted on study identifier, year, design, setting, population, sample size, CBT delivery format, comparator, outcome definition, intervention and control events, and intervention and control non-events. For smoking abstinence, an event was defined as complete cessation at follow-up. For smoking reduction, an event was defined as the study-reported clinically meaningful reduction in cigarette or tobacco consumption without necessarily achieving full abstinence. When a study reported percentages or proportions, these values were converted to event counts using the available denominators; when a zero cell occurred, a continuity correction of 0.5 was applied to all four cells for that study-specific comparison. A narrative synthesis and tabulation of study characteristics were conducted before quantitative pooling.

### Statistical analysis

The common effect metric was the odds ratio (OR), with values above 1.00 favoring CBT over control conditions. Separate DerSimonian and Laird random-effects meta-analyses were conducted for smoking abstinence and smoking reduction because clinical and methodological heterogeneity was expected across CBT protocols, populations, comparator intensity, and follow-up definitions^13^. Statistical heterogeneity was assessed^14^ using Cochran’s Q, reported with degrees of freedom, the I^2^ statistic, and tau-squared. A two-sided p<0.05 was considered statistically significant. Analyses were conducted in Python 3.11 using *NumPy*, *pandas*, *SciPy*, and *Matplotlib*. Publication bias was assessed for outcomes with at least 10 studies using visual inspection of funnel plots and Egger’s regression^15^.

Exploratory sensitivity analyses included fixed-effect pooling, randomized-trial-only pooling, and leave-one-out analyses. Formal subgroup analyses by delivery format, population type, CBT intensity, and comparator type were limited by the small number of studies and sparse reporting; therefore, these analyses were interpreted descriptively rather than as confirmatory tests.

## RESULTS

### Study characteristics and extracted data

**Figure 1:**
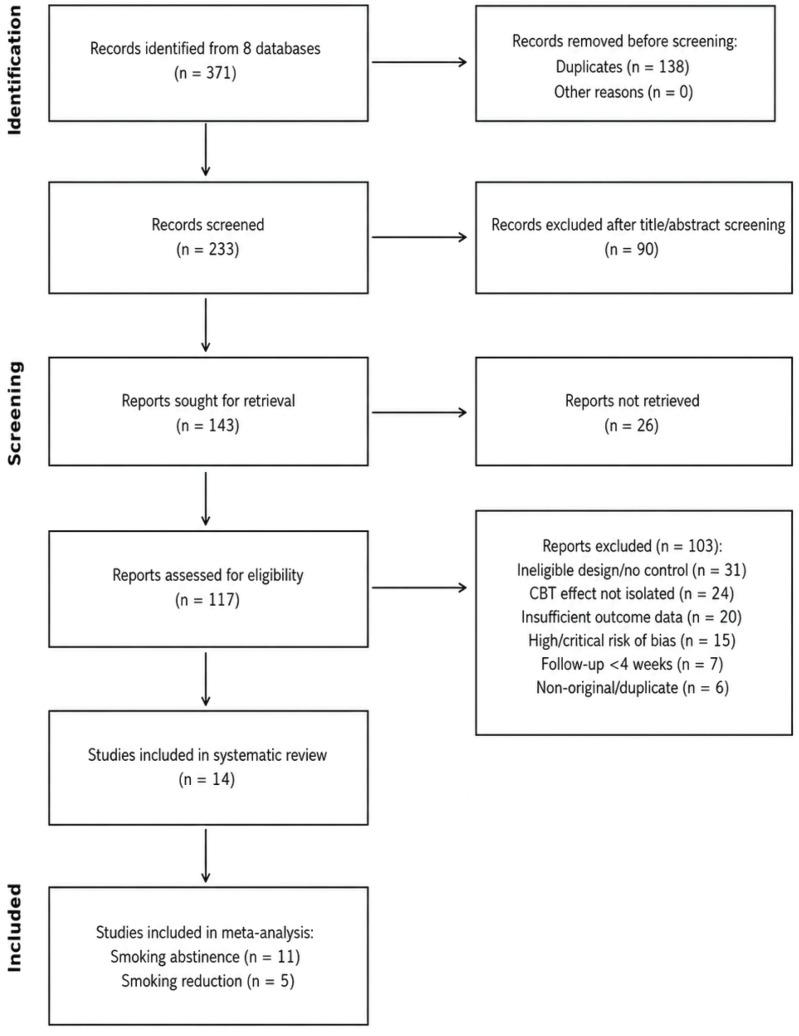
PRISMA flow diagram for the systematic review of cognitive behavioral therapy for smoking cessation. The diagram shows the search in 8 databases, from 1 January 2000 to 15 January 2026, reasons for excluding 103 full-text reports, and the number of studies included in each meta-analysis model

The search yielded 371 records. After the removal of 138 duplicates, 233 records were screened. Ninety records were excluded during title and abstract screening, and 143 reports were sought for full-text retrieval. Twenty-six reports could not be retrieved, leaving 117 full texts for eligibility assessment. A further 103 reports were excluded because of an ineligible design or absence of a control group, inability to isolate the effect of CBT, insufficient outcome data, high or critical risk of bias, follow-up shorter than four weeks, or non-original/duplicate publication status. Fourteen studies were included in the review and meta-analysis (Figure 1).

Fourteen studies met the eligibility criteria and contributed data to the quantitative synthesis^16-29^. Eleven studies reported smoking abstinence outcomes, and five reported smoking reduction outcomes; Baker et al.^16^ and Selvamary et al.^29^ contributed data to both outcomes. Study characteristics, including design, setting, sample size, population, intervention, and comparator, are presented in Table 1. The extracted 2×2 data used for effect-size calculation are presented in Table 2.

**Table 1:** Characteristics of the included controlled studies of cognitive behavioral therapy for smoking cessation from 1 January 2000 to 15 January 2026 (N=14)

*Study*	*Design*	*Setting/population*	*Sample* *size*	*CBT delivery*	*Comparator*	*Outcomes* *contributed*
Baker et al.^16^ 2006	Randomized controlled trial	Psychotic disorder/mental-health setting; People with a psychotic disorder who smoked	298	Face-to-face CBT plus cessation support	Usual care/brief advice	abstinence, reduction
Castaldelli-Maia et al.^17^ 2018	Controlled observational study	Real-world cessation treatment service; People with and without mental or substance use disorders	696	Clinic-based behavioral/CBT-oriented treatment	Non-CBT or less intensive support	abstinence
Castaldelli-Maia et al.^18^ 2013	Controlled observational study	Addiction care unit; People receiving smoking cessation treatment	367	Clinic-based behavioral/CBT-oriented treatment	Non-CBT or less intensive support	abstinence
Evins et al.^19^ 2001	Pilot randomized trial	Schizophrenia treatment setting: People with schizophrenia who smoked	18	CBT with pharmacotherapy support	Control condition	reduction
Farooq et al.^20^ 2020	Randomized controlled trial	Public-health/dental setting; Adults who smoked	60	Cognitive behavioral therapy	Basic health education	reduction
Hajisahneh et al.^21^ 2025	Controlled intervention trial	Community/relapse-prevention setting; Iranian people who smoked	235	Cognitive behavioral relapse-prevention intervention	Control condition	abstinence
Hendricks et al.^22^ 2010	Randomized controlled trial	Tobacco-dependence treatment setting: People receiving extended treatment for tobacco dependence	199	Extended CBT for tobacco dependence	Standard cessation treatment	abstinence
Johnson et al.^23^ 2026	Pilot randomized clinical trial	Smoking cessation trial setting: People seeking smoking cessation treatment	82	CBT-supported cessation program	Active comparator	abstinence
Martínez-Vispo et al.^24^ 2019	Randomized controlled trial	Smoking cessation treatment setting: Adults who smoked	219	CBT with behavioral activation	Standard CBT/ control condition	abstinence
Morean et al.^25^ 2015	Randomized controlled trial	Adolescent smoking cessation trial: Highly impulsive adolescent smokers	64	Cognitive behavioral therapy	Contingency management	abstinence
Mueller et al.^26^ 2012	Randomized controlled trial	Alcohol detoxification treatment setting: People receiving alcohol detoxification treatment who smoked	103	CBT smoking cessation during detoxification	Control condition	reduction
Park et al.^27^ 2014	Randomized comparative trial	Nicotine dependence treatment setting: People with nicotine dependence	30	Cognitive behavioral therapy	Virtual cue exposure therapy	abstinence
Rovina et al.^28^ 2009	Controlled clinical-practice study	Respiratory clinical-practice setting; People receiving smoking cessation treatment in clinical practice	71	Behavioral/CBT-oriented cessation intervention	Pharmacotherapy or behavioral comparator	abstinence
Selvamary et al.^29^ 2016	Randomized controlled trial	Hospital dental setting; People using tobacco in a dental setting	194	Cognitive behavior therapy	Basic advice/ control support	abstinence, reduction

CBT: cognitive behavioral therapy. Sample size indicates the total number of participants represented in the extracted 2×2 outcome data.

**Table 2:** Extracted outcome data and odds ratios for smoking abstinence and smoking reduction in the included studies, with outcome definitions used for synthesis

*Study*	*Outcome*	*Outcome definition used for* *synthesis*	*Treatment* *events*	*Treatment* *non-events*	*Control* *events*	*Control* *non-events*	*OR*	*95 % CI*
Baker et al.^16^ 2006	abstinence	Complete abstinence at follow-up	27	120	10	141	3.17	1.48–6.82
Baker et al.^16^ 2006	reduction	Study-defined smoking reduction among continuing smokers	46	101	27	124	2.09	1.22–3.60
Castaldelli-Maia et al.^17^ 2018	abstinence	Complete abstinence at follow-up	86	191	144	275	0.86	0.62–1.19
Castaldelli-Maia et al.^18^ 2013	abstinence	Complete abstinence at follow-up	68	80	69	150	1.85	1.20–2.84
Evins et al.^19^ 2001	reduction	Study-defined reduction in smoking; threshold varied across reports	6	3	1	8	16.00	1.32–194.63
Farooq et al.^20^ 2020	reduction	Study-defined tobacco-use reduction at follow-up	4	26	1	29	4.46	0.47–42.52
Hajisahneh et al.^21^ 2025	abstinence	Complete abstinence at follow-up	91	21	116	7	0.26	0.11–0.64
Hendricks et al.^22^ 2010	abstinence	Complete abstinence at follow-up	54	45	34	66	2.33	1.31–4.13
Johnson et al.^23^ 2026	abstinence	Complete abstinence at follow-up	17	25	4	36	6.12	1.84–20.38
Martínez-Vispo et al.^24^ 2019	abstinence	Complete abstinence at follow-up	71	39	50	59	2.15	1.25–3.70
Morean et al.^25^ 2015	abstinence	Complete abstinence at follow-up	0	22	15	27	0.04	0.00–0.70
Mueller et al.^26^ 2012	reduction	Study-defined reduction in cigarette consumption	17	36	8	42	2.48	0.96–6.42
Park et al.^27^ 2014	abstinence	Complete abstinence at follow-up	10	5	11	4	0.73	0.15–3.49
Rovina et al.^28^ 2009	abstinence	Complete abstinence at follow-up	10	25	7	29	1.66	0.55–5.00
Selvamary et al.^29^ 2016	abstinence	Complete abstinence at follow-up	64	30	30	70	4.98	2.71–9.15
Selvamary et al.^29^ 2016	reduction	Study-defined tobacco-use reduction at follow-up	51	43	75	25	0.40	0.22–0.73

OR: odds ratio. CI: confidence interval. Treatment events and control events indicate participants achieving the specified outcome. A continuity correction = 0.5 was applied to cells for comparisons with a zero event count.

### Heterogeneity and sensitivity analyses

Substantial heterogeneity was observed for both outcomes (Table 3). For smoking abstinence, Q=61.36 (df=10, p<0.001), I^2^=83.70%, and τ^2^=0.56. For smoking reduction, Q=24.44 (df=4, p<0.001), I^2^=83.64%, and τ^2^=1.09.

**Table 3:** Heterogeneity, random-effects pooled estimates, and sensitivity analyses for smoking abstinence and smoking reduction in controlled studies of cognitive behavioral therapy for smoking cessation

*Analysis*	*Outcome*	*Number of* *studies*	*Pooled OR*	*95% CI*	*Q (df)*	*I^2^ (%)*	*p*
Primary random-effects	abstinence	11	1.60	0.95–2.70	61.36 (10)	83.70	0.078
Fixed-effect sensitivity	abstinence	11	1.56	1.29–1.87	Not applicable	Not applicable	<0.001
Primary random-effects	reduction	5	1.96	0.66–5.81	24.44 (4)	83.64	0.226
Fixed-effect sensitivity	reduction	5	1.26	0.87–1.81	Not applicable	Not applicable	0.216
Randomized-trial-only sensitivity	abstinence	8	1.66	0.79–3.46	41.65 (7)	83.19	0.180
Leave-one-out excluding Selvamary et al.^29^ 2016	reduction	4	2.40	1.52–3.77	Not applicable	0.00	<0.001

OR: odds ratio. CI: confidence interval. df: degrees of freedom. I^2^: inconsistency statistic. Random-effects estimates used the DerSimonian and Laird method.

Leave-one-out analyses showed that the direction and precision of the abstinence estimate were sensitive to individual studies: excluding Castaldelli-Maia et al.^17^, Hajisahneh et al.^21^, or Morean et al.^25^ moved the random-effects abstinence estimate to statistical significance, whereas excluding several studies with favorable CBT effects moved it further toward the null. For smoking reduction, excluding Selvamary et al.^29^ changed the pooled estimate to OR=2.40 (95% CI: 1.52–3.77) and reduced I^2^ to 0.0%, indicating that this study was the main driver of heterogeneity in the reduction analysis. The randomized/controlled-trial-only sensitivity analysis for abstinence produced OR=1.66 (95% CI: 0.79–3.46), supporting a favorable but imprecise direction of effect.

### Smoking abstinence

**Figure 2:**
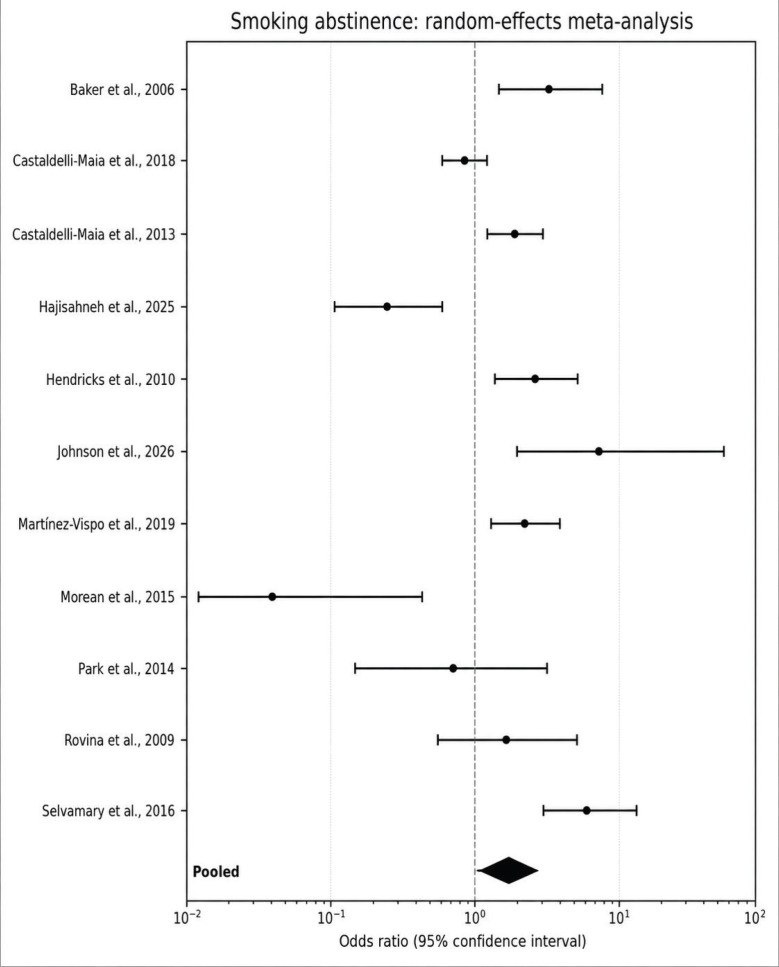
Forest plot of odds ratios and 95% confidence intervals for smoking abstinence in eleven controlled studies of cognitive behavioral therapy for smoking cessation. The pooled diamond represents the DerSimonian and Laird random-effects estimate with values >1 favoring cognitive behavioral therapy

The meta-analysis of smoking abstinence included 11 studies. The random-effects pooled OR was 1.60 (95% CI: 0.95–2.70; z=1.76; p=0.078), indicating a favorable but not statistically conclusive association between CBT and complete smoking abstinence (Figure 2). A fixed-effect sensitivity analysis yielded OR=1.56 (95% CI: 1.29–1.87; p<0.001), but the random-effects estimate was considered more appropriate because heterogeneity was substantial.

### Smoking reduction

**Figure 3:**
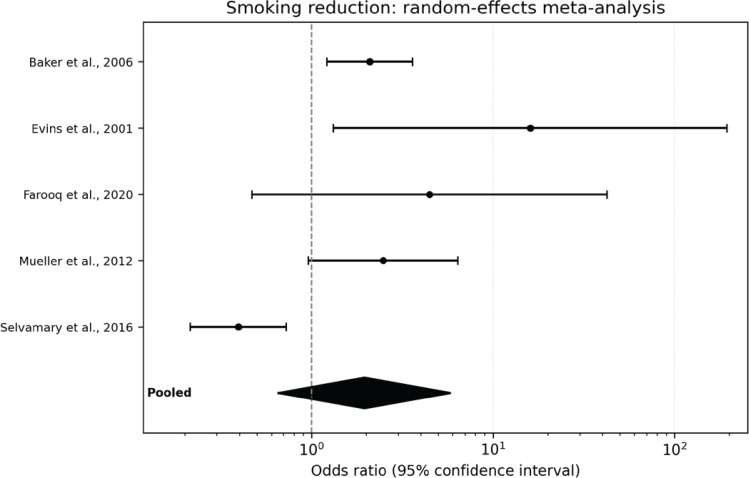
Forest plot of odds ratios and 95% confidence intervals for smoking reduction in five controlled studies of cognitive behavioral therapy for smoking cessation. The pooled diamond represents the DerSimonian and Laird random-effects estimate with values >1 favoring cognitive behavioral therapy

The smoking reduction analysis included five studies. The random-effects pooled OR was 1.96 (95% CI: 0.66–5.81; z=1.21; p=0.226), indicating no statistically conclusive advantage of CBT over control conditions for reducing cigarette or tobacco consumption (Figure 3). The study-specific definitions of reduction varied and were not consistently reported with comparable thresholds, so this outcome should be interpreted cautiously.

### Risk-of-bias

Risk-of-bias assessment identified low risk or some concerns for most randomized trials and moderate risk for the controlled observational studies. Common reasons for some concerns included incomplete reporting of allocation procedures, lack of blinding for behavioral interventions, attrition, and incomplete information on prespecified outcome reporting. No included study was judged to have high or critical risk of bias in key domains (Table 4).

**Table 4:** Risk-of-bias assessment for the included controlled studies of cognitive behavioral therapy for smoking cessation (N=14)

*Study*	*Design/tool*	*Randomization* *or confounding*	*Deviations* *from intended* *intervention*	*Missing* *outcome* *data*	*Outcome* *measurement/* *reporting*	*Overall* *judgment*
Baker et al.^16^ 2006	RCT/RoB 2	Some concerns	Low risk	Some concerns	Low risk	Some concerns
Castaldelli-Maia et al.^17^ 2018	Non-randomized/ROBINS–I	Moderate risk of confounding	Low/moderate	Moderate	Low/moderate	Moderate risk
Castaldelli-Maia et al. ^18^ 2013	Non-randomized/ROBINS–I	Moderate risk of confounding	Low/moderate	Moderate	Low/moderate	Moderate risk
Evins et al.^19^ 2001	Pilot RCT/RoB 2	Some concerns	Low risk	Some concerns	Low risk	Some concerns
Farooq et al.^20^ 2020	RCT/RoB 2	Some concerns	Low risk	Low risk	Some concerns	Some concerns
Hajisahneh et al.^21^ 2025	Controlled trial/RoB 2	Some concerns	Low risk	Low risk	Some concerns	Some concerns
Hendricks et al.^22^ 2010	RCT/RoB 2	Low risk	Low risk	Some concerns	Low risk	Some concerns
Johnson et al.^23^ 2026	Pilot RCT/RoB 2	Some concerns	Low risk	Some concerns	Low risk	Some concerns
Martínez-Vispo et al.^24^ 2019	RCT/RoB 2	Low risk	Low risk	Low risk	Low risk	Low risk
Morean et al.^25^ 2015	RCT/RoB 2	Some concerns	Low risk	Some concerns	Low risk	Some concerns
Mueller et al.^26^ 2012	RCT/RoB 2	Some concerns	Low risk	Some concerns	Low risk	Some concerns
Park et al.^27^ 2014	Randomized comparative trial/RoB 2	Some concerns	Low risk	Some concerns	Some concerns	Some concerns
Rovina et al.^28^ 2009	Non-randomized/ROBINS–I	Moderate risk of confounding	Low/moderate	Moderate	Low/moderate	Moderate risk
Selvamary et al.^29^ 2016	RCT/RoB 2	Some concerns	Low risk	Low risk	Some concerns	Some concerns

RoB 2: revised Cochrane Risk-of-Bias 2 tool. ROBINS–I: risk-of-bias in non-randomized studies of interventions. No included study was judged to have a high or critical risk of bias in key domains.

### Publication bias

**Figure 4:**
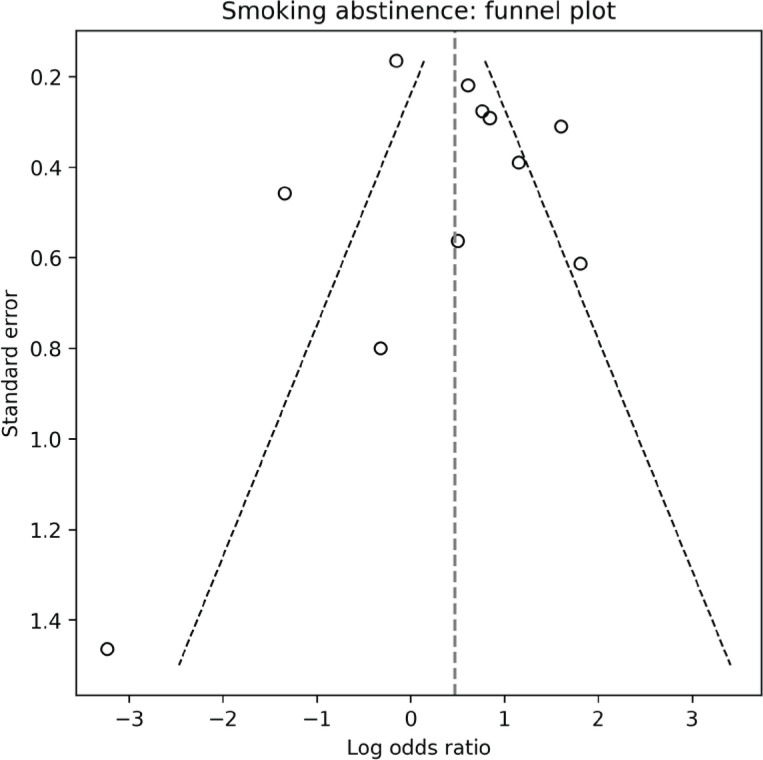
Funnel plot for publication bias assessment of smoking abstinence in eleven controlled studies of cognitive behavioral therapy for smoking cessation. The horizontal axis shows the log odds ratio, the vertical axis shows the standard error, and the dashed lines indicate the pooled effect and approximate 95% confidence interval region

Publication bias was assessed quantitatively for smoking abstinence because 11 studies were available. The funnel plot showed visual asymmetry (Figure 4), and Egger’s regression produced an intercept of 3.041 with p=0.111. This result did not provide statistically compelling evidence of small-study effects, although the modest number of studies limits inference. Publication bias for smoking reduction could not be tested quantitatively because fewer than 10 studies were available, but selective publication remains possible because small studies with null reduction findings may be less likely to be published.

## DISCUSSION

### Principal findings

This systematic review and meta-analysis found a favorable direction of effect for CBT on smoking abstinence, but the random-effects estimate was imprecise and did not reach the prespecified statistical significance threshold. The pooled effect for smoking reduction was also not statistically conclusive. These findings suggest that CBT may be more closely aligned with complete cessation than with reduced cigarette consumption, but the strength of this inference is limited by substantial between-study heterogeneity.

The observed distinction between abstinence and reduction outcomes is clinically plausible. Standard CBT for smoking cessation is designed to restructure smoking-related cognitions, strengthen self-efficacy, anticipate triggers, and build coping responses for high-risk situations^4,5^. These mechanisms may be particularly relevant when individuals aim to stop smoking completely and adopt a non-smoking identity. By contrast, smoking reduction may require additional or different components, such as controlled tapering, medication-supported reduction, or reinforcement-based strategies, which may not be central to all CBT protocols.

For clinical practice, these findings support considering CBT as one component of cessation services for people who are motivated to achieve complete abstinence, rather than as a uniformly effective stand-alone intervention across all settings. For people who are not ready to quit completely, CBT alone may be insufficient and may need to be combined with pharmacotherapy, motivational approaches, or contingency management. Because the included studies varied in delivery format, comparator intensity, population, and outcome definitions, implementation should be tailored to the clinical context and cessation goal.

### Strengths and limitations

This review has several strengths. It focused on controlled studies in which CBT was a primary intervention component, separated abstinence from reduction outcomes, converted effects to clinically interpretable ORs, assessed risk of bias, explored heterogeneity, and conducted leave-one-out sensitivity analyses.

Several limitations should also be acknowledged. First, heterogeneity was substantial for both outcomes, reflecting differences in CBT content, session number, delivery format, comparator intensity, population characteristics, and outcome definitions. The pooled estimates should therefore be interpreted as average effects rather than effects expected in every setting. Second, subgroup analyses by delivery format, population type, CBT intensity, and comparator type were limited by sparse and uneven reporting. Third, only five studies reported smoking reduction, limiting statistical power and preventing robust assessment of publication bias for that outcome. Fourth, restricting inclusion to English-language peer-reviewed publications may have introduced language and publication bias. Fifth, some multimodal interventions were excluded to isolate CBT effects, which improves internal interpretability but may reduce generalizability to real-world cessation programs where CBT is commonly combined with pharmacotherapy or other behavioral strategies. Finally, aggregate data limited the exploration of individual participant-level moderators.

### Future research

Future trials should define abstinence and reduction outcomes prospectively, use biochemical verification where feasible, and report sufficient data for meta-analysis. Research should examine whether treatment response varies by nicotine dependence, motivation to quit, psychiatric comorbidity, socioeconomic status, age, gender, and delivery format. Studies comparing standard CBT, enhanced CBT, digital CBT, and CBT combined with pharmacotherapy would help identify the most effective and cost-effective approaches. Individual participant data meta-analyses could further clarify who benefits most from CBT and for which cessation goals.

## CONCLUSIONS

CBT showed a favorable direction of effect for smoking abstinence, but the primary random-effects estimate was imprecise, and heterogeneity was substantial. Evidence for smoking reduction was not statistically conclusive, suggesting that harm-reduction goals may require different or additional intervention components. Future research should refine CBT protocols, standardize outcome definitions, evaluate moderators of response, and address heterogeneity across populations and delivery settings.

## Supplementary Material



## Data Availability

All data analyzed in this review are available from the cited publications.
