# Supplementary material for: Efficacy of cognitive behavioral therapy for smoking cessation: A systematic review and meta-analysis
Source: Tob Induc Dis. 2026 Jul 22;24:10.18332/tid/225228. doi: 10.18332/tid/225228 (PMC13401256; doi:10.18332/tid/225228)
Supplement: Supplementary file 1 [file TID-24-120-s1.pdf]

Tobacco Induced Diseases

Supplementary file

© 2026 Okawa Y.

DOI: 10.18332/tid/225228

The content has been provided by the author(s) and has not been reviewed, verified, or endorsed by European Publishing. It may not have undergone peer review. The views, opinions, and recommendations expressed are solely those of the author(s) and do not necessarily reflect the position of European Publishing. European Publishing accepts no responsibility or liability for any consequences arising from the use of, or reliance on, this content.

Supplementary file Table 1. Complete database-specific search strategies were used to identify studies of cognitive behavioral therapy for smoking cessation from 1 January 2000 to 15 January 2026

**Database**	**Search strategy**	**Limits/notes**
PubMed	(("cognitive behavioral therapy"[Title/Abstract] OR "cognitive behavioural therapy"[Title/Abstract] OR CBT[Title/Abstract] OR "Cognitive Behavioral Therapy"[MeSH Terms]) AND ("smoking cessation"[Title/Abstract] OR "tobacco cessation"[Title/Abstract] OR "Smoking Cessation"[MeSH Terms] OR "quit smoking"[Title/Abstract] OR "smoking abstinence"[Title/Abstract]))	Publication dates: 2000/01/01 to 2026/01/15; humans
Web of Science	TS=(("cognitive behavioral therapy" OR "cognitive behavioral therapy" OR CBT OR "cognitive therapy") AND ("smoking cessation" OR "tobacco cessation" OR "quit smoking" OR "smoking abstinence"))	Timespan 2000-2026; article/document types screened manually
Scopus	TITLE-ABS-KEY(("cognitive behavioral therapy" OR "cognitive behavioral therapy" OR CBT OR "cognitive therapy") AND ("smoking cessation" OR "tobacco cessation" OR "quit smoking" OR "smoking abstinence"))	Publication years 2000-2026
ScienceDirect	("cognitive behavioral therapy" OR "cognitive behavioral therapy" OR CBT OR "cognitive therapy" OR "behavioral therapy" OR "behavioral therapy") AND ("smoking cessation" OR "tobacco cessation" OR "quit smoking" OR "smoking abstinence")	Research articles; 2000-2026
SpringerLink	("cognitive behavioral therapy" OR "cognitive behavioral therapy" OR CBT OR "cognitive therapy" OR "behavioral therapy" OR "behavioral therapy") AND ("smoking cessation" OR "tobacco cessation" OR "quit smoking" OR "smoking abstinence")	Article/chapter records screened; empirical controlled studies retained
ACM Digital Library	("cognitive behavioral therapy" OR "cognitive behavioral therapy" OR CBT OR "cognitive therapy" OR "behavioral therapy" OR "behavioral therapy") AND ("smoking cessation" OR "tobacco cessation" OR "quit smoking" OR "smoking abstinence")	Digital or technology-delivered CBT records screened
IEEE Xplore	("cognitive behavioral therapy" OR "cognitive behavioral therapy" OR CBT OR "cognitive therapy" OR "behavioral therapy" OR "behavioral therapy") AND ("smoking cessation" OR "tobacco cessation" OR "quit smoking" OR "smoking abstinence")	Digital, mobile, or telehealth intervention records were screened
Google Scholar	("cognitive behavioral therapy" OR "cognitive behavioral therapy" OR CBT OR "cognitive therapy" OR "behavioral therapy" OR "behavioral therapy") AND ("smoking cessation" OR "tobacco cessation" OR "quit smoking" OR "smoking abstinence")	First 200 relevance-ranked records screened; records outside date range or without controlled outcome data excluded

Note. Search terms were adapted to the database syntax. MeSH terms were used in PubMed where appropriate.
Searches were supplemented by manual screening of reference lists of eligible reports and relevant reviews.
